# Molecular insights into biochar-mediated plant growth promotion and systemic resistance in tomato against Fusarium crown and root rot disease

**DOI:** 10.1038/s41598-020-70882-6

**Published:** 2020-08-18

**Authors:** Amit K. Jaiswal, Noam Alkan, Yigal Elad, Noa Sela, Amit M. Philosoph, Ellen R. Graber, Omer Frenkel

**Affiliations:** 1 Department of Plant Pathology and Weed Research, Institute of Plant Protection, The Volcani Center (ARO), 7505101 Rishon Lezion, Israel; 2 Department of Soil Chemistry, Plant Nutrition and Microbiology, Institute of Soil, Water and Environmental Sciences, The Volcani Center (ARO), 7505101 Rishon Lezion, Israel; 3 Department of Postharvest Science of Fresh Produce, Institute of Plant Harvest and Food Sciences, The Volcani Center (ARO), 7505101 Rishon Lezion, Israel; 4 Department of Plant Pathology and Microbiology, The Robert H. Smith Faculty of Agriculture, Food and Environment, The Hebrew University of Jerusalem, 761001 Rehovot, Israel; 5 Department of Horticulture and Landscape Architecture, Purdue University, West Lafayette, IN USA

**Keywords:** Biotic, Fungi

## Abstract

Molecular mechanisms associated with biochar-elicited suppression of soilborne plant diseases and improved plant performance are not well understood. A stem base inoculation approach was used to explore the ability of biochar to induce systemic resistance in tomato plants against crown rot caused by a soilborne pathogen, *Fusarium oxysporum* f. sp. *radicis lycopersici.* RNA-seq transcriptome profiling of tomato, and experiments with jasmonic and salycilic acid deficient tomato mutants, were performed to elucidate the *in planta* molecular mechanisms involved in induced resistance. Biochar (produced from greenhouse plant wastes) was found to mediate systemic resistance against Fusarium crown rot and to simultaneously improve tomato plant growth and physiological parameters by up to 63%. Transcriptomic analysis (RNA-seq) of tomato demonstrated that biochar had a priming effect on gene expression and upregulated the pathways and genes associated with plant defense and growth such as jasmonic acid, brassinosteroids, cytokinins, auxin and synthesis of flavonoid, phenylpropanoids and cell wall. In contrast, biosynthesis and signaling of the salicylic acid pathway was downregulated. Upregulation of genes and pathways involved in plant defense and plant growth may partially explain the significant disease suppression and improvement in plant performance observed in the presence of biochar.

## Introduction

Biochar (the solid co-product of biomass pyrolysis) is a carbon sequestrating soil amendment reported to improve plant performance and reduce the severity of both foliar and soilborne plant diseases^1,2^. Nevertheless, results are highly dependent on the biochar dose, feedstock, production conditions and pathosystems^2–5^. The variability in plants responses and a poor understanding of the mechanisms involved in plant growth promotion and disease suppression are among the factors hampering the widespread adoption of biochar as a beneficial soil amendment.

Biochar amendment-elicited suppression of diseases caused by foliar pathogens is clearly mediated by induced systemic resistance, given that biochar is spatially distant from the site of pathogen attack^6–9^. In contrast, there are numerous means by which biochar may influence diseases caused by soilborne plant pathogens. Biochar and pathogens both reside in the soil and thus biochar can potentially have direct antagonistic effects toward the pathogen^10,11^ as well as indirect interactions via induction of systemic resistance in the plant^10–12^. Induction of the plant’s innate defense system can decrease its susceptibility to diseases caused by a broad range of pathogens and parasites, including soilborne pathogens^13^. Two major forms of induced resistance (IR) have been described in plants: induced systemic resistance (ISR) and systemic acquired resistance (SAR). ISR, commonly attributed to plant growth-promoting rhizobacteria (PGPR) and fungi (PGPF), depends on the phytohormones ethylene (ET), and jasmonic acid (JA)^14,15^. SAR, which can be triggered by both chemical and biological elicitors, involves synthesis of pathogenesis-related proteins and is mediated by the phytohormone salicylic acid (SA)^16–18^. Phytohormones abscisic acid (ABA), gibberellic acid (GA), cytokine (CK), auxin (IAA), and brassinosteroids (BR) that are typically associated with abiotic stress or plant developmental processes, are also involved in plant immunity and growth/development in plants^19^. Elicitors of biochar-potentiated plant defenses include biochar-borne chemicals and biochar-induced microorganisms^10,12,20^.

Global gene expression (microarrays) data for *Arabidopsis thaliana* grown in soil amended with biochar (4.2% w:w high temperature gasification biochar) revealed the up-regulation of several genes involved in stimulating plant growth and concomitant down-regulation of defense-related genes^21^. Yet, since no pathogen challenge was made^21^, it is not known whether the reported general downregulation of defense-involved genes would have resulted in subsequent susceptibility to pathogen attack. So far, evidence for induction of systemic plant defenses by biochar has been presented only for foliar diseases such as *Botrytis cinerea*—gray mold and *Podosphaera apahanis*—powdery mildew in strawberry^9^, *B. cinerea*—gray mold in tomato^8^, and *Rhizoctonia*—foliar blight in soybean^22^, through observing several genes by qRT-PCR. Molecular mechanisms associated with biochar-elicited suppression of soilborne plant diseases have not yet been documented.

The current study addresses these knowledge gaps using the tomato (*Solanum lycopersicum*) and soilborne fungal pathogen *Fusarium oxysporum* f. sp. *radicis lycopersici* Jarvis and Shoemaker (FORL) system. This soilborne Ascomycete, the causal agent of Fusarium crown and root rot (FCRR), is a destructive soilborne pathogen of tomato in greenhouse and field production, reducing yields by 15–65%^23^. The goal is to decipher the molecular pathways and genes involved in improved plant performance and induced systemic resistance against a soilborne disease. To the best of our knowledge, there has not yet been a comprehensive study that includes a whole transcriptomic profile of plants grown in biochar that were subjected to either foliar or soilborne pathogen attack.

## Results

### Effect of biochar on disease suppression and plant performance: potting mixture inoculation approach

**Figure 1:**
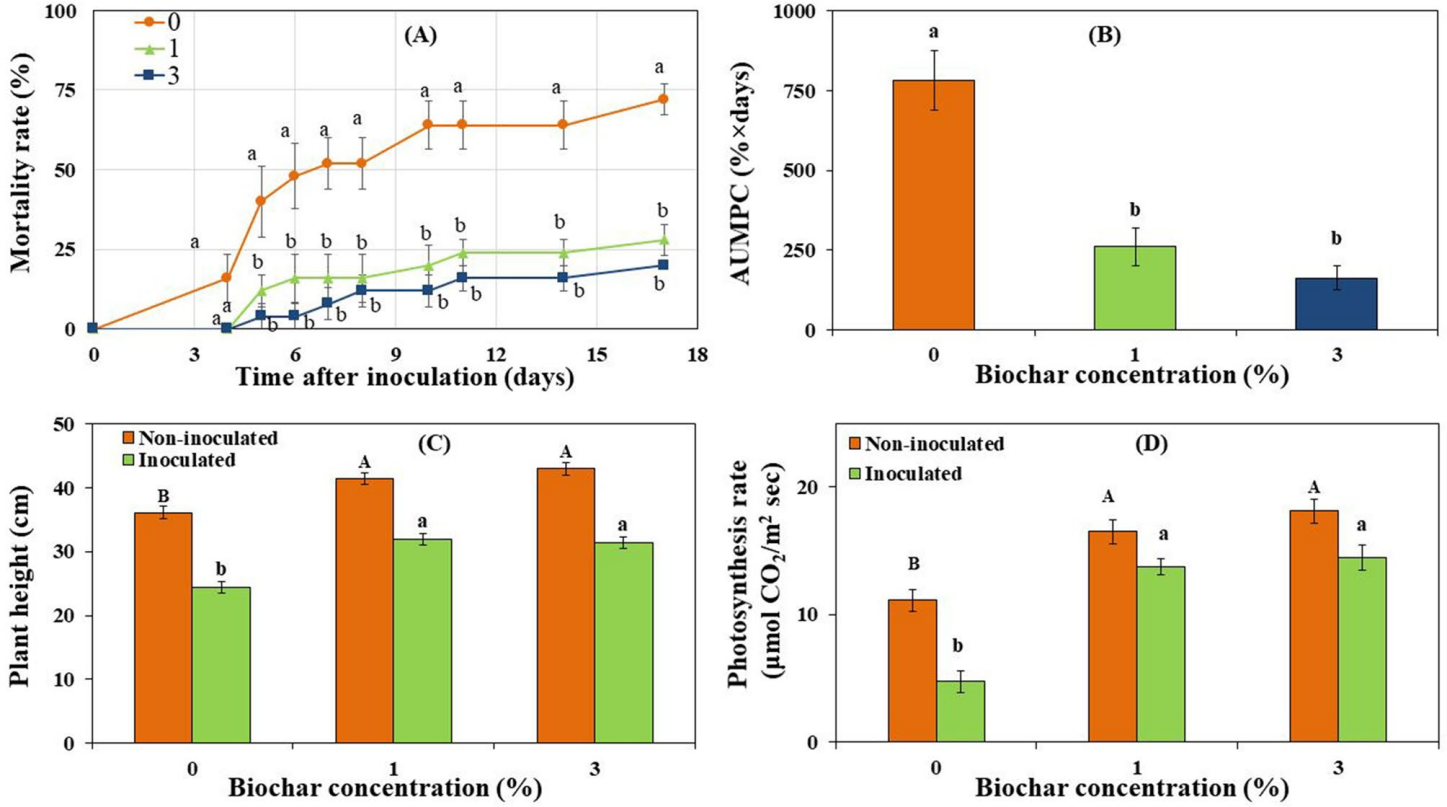
Effect of greenhouse waste (GHW-350) biochar soil amendments at concentrations of 0, 1, and 3% on the (**A**) progress of mortality rates caused by soil-inoculated FORL, (**B**) Area Under the Mortality Progress Curve (AUMPC), (**C**) plant height, and (**D**) photosynthesis rate. Columns or data points labeled with a different letter are significantly different at *P* ≤ 0.05 according to Tukey Kramer HSD test. Bars represent the standard error.

First, the effect of GHW-350 biochar (produced from greenhouse pepper plant wastes at 350 °C) at 0, 1, and 3% concentration on the Fusarium crown and root rot disease of tomato and plant performance was examined in the glasshouse for 24 days under fertigation and irrigation regimes as in previous studies^12^. In the potting mixture inoculated FORL experiment, disease mortality rates in the 1 and 3% biochar treatments were significantly reduced by 61 and 72%, respectively, as compared to the non-amended control (*P* < 0.0001; Fig. 1a). Accordingly, Area Under the Mortality Progress Curve (AUMPC) value was also significantly lowered in 1 and 3% biochar treatments, by 67 and 79%, respectively, as compared with the non-amended control (*P* < 0.0001; Fig. 1b).

Plant height and net photosynthesis rate of the tomato plants had significant positive responses to biochar in both non-inoculated and inoculated treatments. In the absence of the pathogen, biochar significantly stimulated plant growth by 15 and 20% at the 1 and 3% biochar concentrations, respectively, as compared to the non-amended control (*P* < 0.005; Fig. 1c). Similarly, photosynthesis rate was increased by 49 and 63% at 1 and 3% biochar rates, respectively (*P* < 0.001; Fig. 1d). In the presence of the pathogen, biochar enhanced plant growth by 31 and 29% at 1 and 3% biochar concentrations, respectively, compared with the inoculated, no biochar treatment (*P* = 0.0001; Fig. 1c); the photosynthesis rate was significantly increased by 191 and 207% at 1 and 3% biochar, respectively (*P* < 0.0001; Fig. 1d).

### Biochar-elicited induced resistance (IR) to FORL: stem base inoculation approach

**Figure 2:**
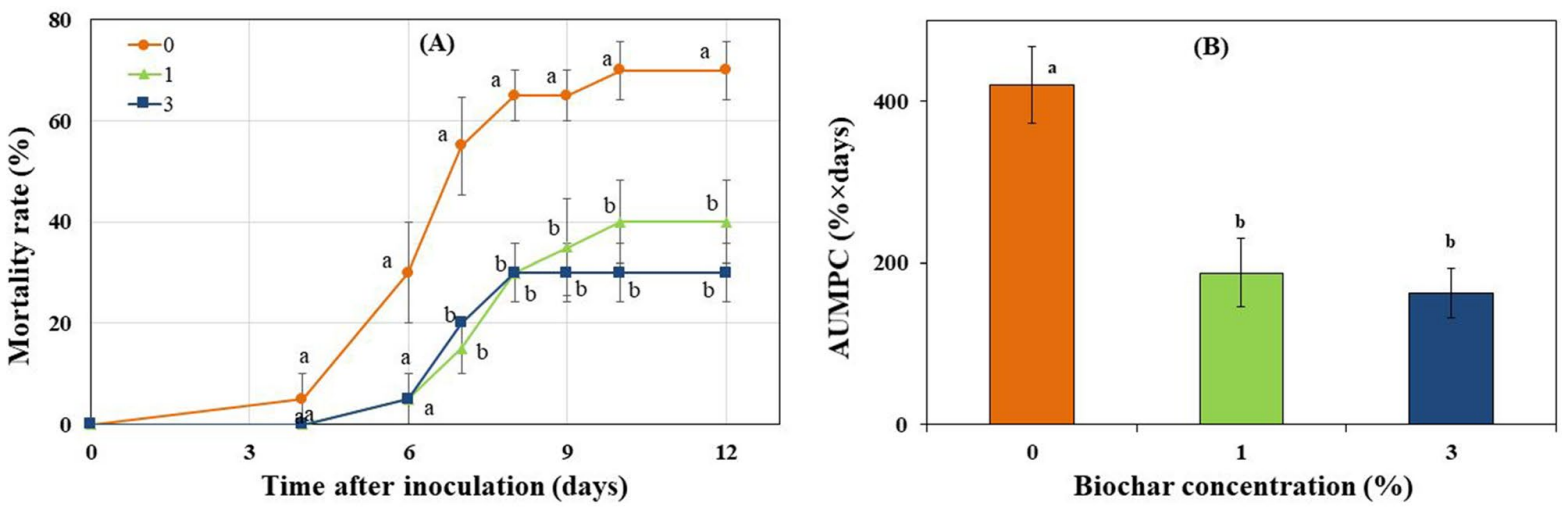
Effect of greenhouse waste (GHW-350) biochar soil amendments at concentrations of 0, 1, and 3% on the (**A**) progress of tomato mortality rates, and (**B**) Area Under the Mortality Progress Curve (AUMPC) caused by stem base-inoculated FORL. Columns or data points labeled with a different letter are significantly different at *P* ≤ 0.05 according to Tukey Kramer HSD test. Bars represent the standard error.

In the potting media inoculation method, biochar and FORL are together in the soil, meaning there are a myriad of direct and indirect ways by which biochar may have influenced the plant responses^10–12^ In order to specifically examine whether biochar may be involved indirectly in induced resistance (IR) of tomato to FORL, an experiment was conducted using a stem base inoculation approach. In this method, the biochar was in the soil and the pathogen was inoculated on the plant stem, spatially separated from the soil. This approach makes it possible to evaluate the indirect effects of biochar on disease through its impact on plant systemic resistance. In the stem inoculation approach, mortality rates of tomato plants were significantly reduced in the biochar treated plants compared with the non-biochar treated plants (Fig. 2a,b), indicating that the presence of biochar mediated a systemic resistance response against FORL. Biochar applied at concentrations of 1 and 3% significantly reduced disease mortality rates by 43 and 57%, respectively, as compared to the non-amended control (*P* < 0.01; Fig. 2a). AUMPC value was also significantly lowered at 1 and 3% biochar by 55 and 61%, respectively, as compared with the non-amended control (*P* < 0.01; Fig. 2b). These reductions were of similar magnitudes to those observed in the conventional potting media inoculation experiment.

### Overview of pathways involved in biochar-elicited IR against FORL using tomato mutants and stem base inoculation method

**Figure 3:**
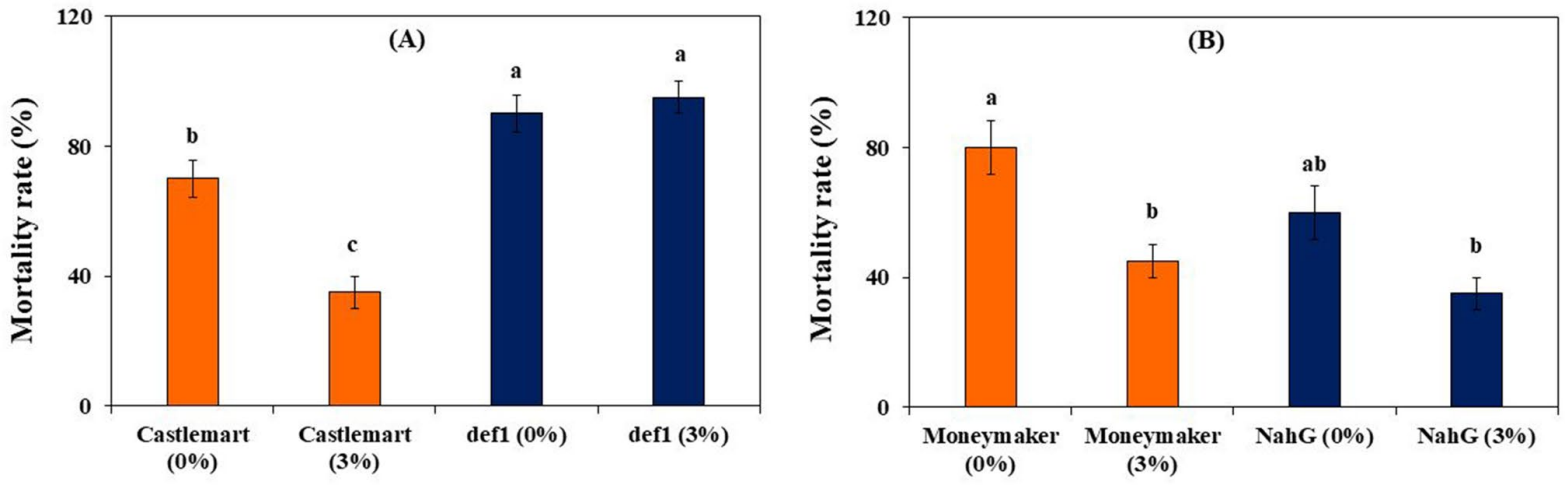
Effect of greenhouse waste (GHW-350) biochar soil amendments at concentrations of 0 and 3% on (**A**) final mortality rates of tomato genotypes cv. Castlemart and its *def1* mutant, and (**B**) final mortality rates of tomato genotypes cv. Moneymaker and its *NahG* transformant. Columns labeled with a different letter are significantly different at *P* ≤ 0.05 according to Tukey Kramer HSD test. Bars represent the standard error.

The contribution of the defense hormones salicylic acid (SA) and jasmonic acid (JA) to biochar-mediated IR was examined using transgenic and mutant plants that were grown in either biochar amended (3% w:w) potting medium or biochar-free potting medium and subsequently inoculated with FORL on the stem base. In the absence of biochar, JA-deficient *def1* mutant was generally more susceptible to FORL than its wild type (WT) Castlemart (Fig. 3a). In contrast, disease incidence in the NahG transgenic line (impaired in SA defense response) was not significantly reduced compared with its WT Moneymaker (Fig. 3b). In biochar treated plants, mortality rate was reduced by about 50% as compared with no biochar treatment in all the genotypes with the exception of the JA-deficient def1 mutant. These results suggest that biochar-mediated IR towards FORL is dependent at least on the JA hormonal pathway.

### Tomato RNA sequence data analysis

To better understand molecular mechanisms involved in the induced systemic resistance and plant growth promotion, mock inoculated control and FORL-inoculated stem base of tomato (M-82 cultivar) grown in biochar (3%) or control media were collected in duplicate at 0, 24, and 72-h post-inoculation (hpi). RNA was extracted and sequenced. A total of 389,038,999 raw reads with an average of 19,451,950 reads per sample with 61 bp in length were obtained from 20 libraries. Low-quality reads were trimmed resulting in an average of 19,179,050 reads, which represent 98.6% high-quality reads. High quality reads were mapped to a tomato reference (ITAG 3.10_cDNA), of which 64% of the reads could be mapped to the tomato genome. Overall, 34,879 transcripts with a mean length of 1,179 bp were identified (Supplementary Table S4).

### Differential expression of genes induced by biochar

**Figure 4:**
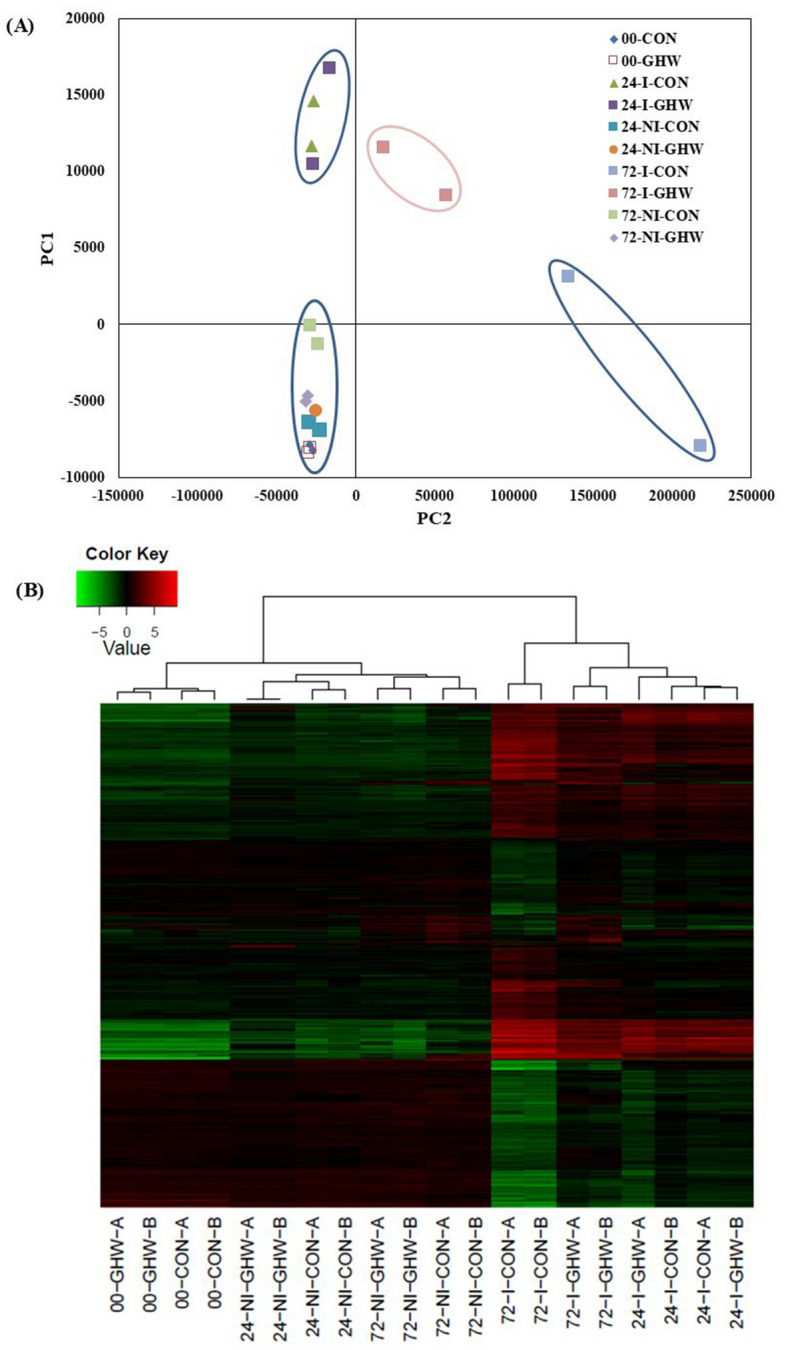
Tomato transcriptomic response to biochar and pathogen inoculation after 0, 24, and 72 h. (**A**) Principal component analysis (PCA). The PC1 and PC2 axis represent 61.1% and 8.3% of the variance respectively and (**B**) Heat map diagram showing the differential expression profiles of 2,464 genes of tomato for three sampling times: 0, 24, and 72 h after inoculation with pathogen (I) or mock (NI) with two biological replicates.

Principal component analysis (PCA) of all expressed genes of tomato showed that the replicates from each treatment were very close to each other. Differences between transcripts expression were calculated using DESeq2 Bioconductor package; we used a threshold of log2 fold change less than − 1 or greater than 1 and FDR less than 0.05. The expression patterns for all mock inoculated samples from biochar and biochar-free treatments at 0, 24, and 72 hpi were relatively similar and clustered together (Fig. 4a). The largest number of DEGs (differentially expressed genes) were found between mock and pathogen inoculated treatments. The transcriptome fingerprint of pathogen-inoculated, biochar and biochar-free treatments, at 24 hpi formed a single cluster, separate from any other clusters. In contrast, at 72 hpi, largest number of DEGs were seen between the two pathogen-inoculated treatments (2,249 genes), with and without biochar (Fig. 4a). There were distinct shifts between the two along both PC1 and PC2 axes, whereby the PC1 axis explains 61.08% of the variance. To examine this change, a hierarchical clustering was further conducted between all the samples. Hierarchical clustering and heat map analysis showed expression patterns of 2,464 genes that were differentially expressed (Fig. 4b). Expression patterns of the pathogen inoculated biochar-amended treatment were different from the pathogen inoculated biochar-free treatment at 24 hpi (11 genes) and a much greater number of DEGs (2,249 genes) at 72 hpi, indicating that the biochar treatment had a priming effect on gene expression upon infection (Table S4).

**Figure 5:**
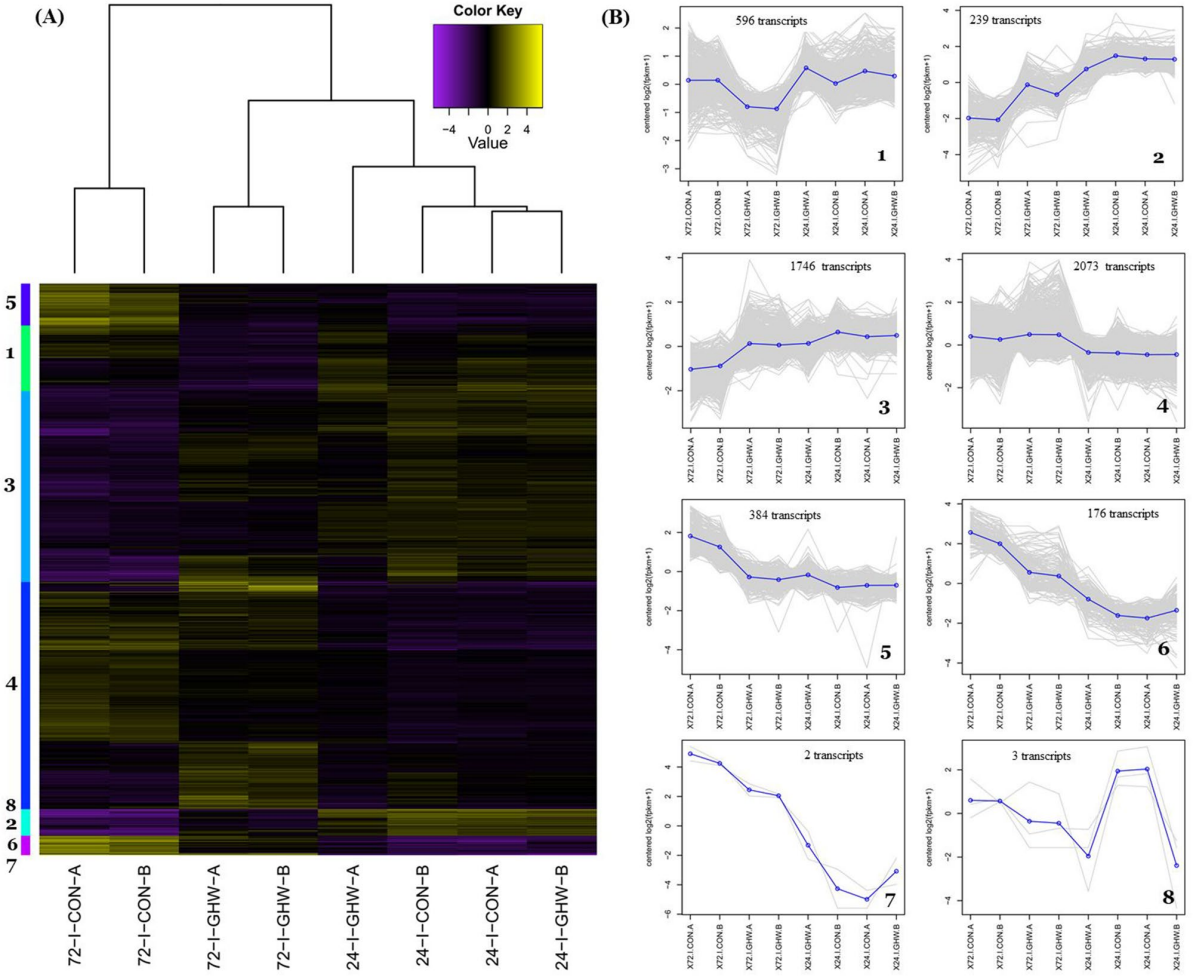
Tomato transcriptomic of inoculated treatments at 24 and 72 hpi. (**A**) Heat map diagram showing differential expression profiles of tomato transcripts at 24 and 72 h after inoculation with FORL. (**B**) Expression patterns of eight clusters of co-expressed differential genes. Each plot is marked with their cluster number each transcript is plotted in gray, in addition to the mean expression profile (blue).

As transcripts of pathogen-inoculated treatments at 24 and 72 hpi were regulated, transcripts from those treatments were further grouped into eight co-expressed clusters according to their differential expression patterns (Fig. 5a,b). At 24 hpi, only 11 transcripts were differentially expressed. At 72 hpi, 2,249 transcripts were differentially expressed, of which 1,193 were upregulated and 1,056 were downregulated (FDR < 0.05, Supplementary Table S4). Since the massive changes in gene regulation occurred only for pathogen-inoculated treatments (i.e. pathogen inoculated biochar-amended and pathogen inoculated biochar-free treatments) at 72 hpi (i.e. 2,249 vs. 11 transcripts differentially expressed at 72 hpi and 24 hpi, respectively), we further characterized the GO-terms, metabolic and signaling pathways only for pathogen-inoculated biochar treatment at 72 hpi. Comparative analysis of regulated transcripts for biochar-amended compared to biochar-free treatment showed upregulation expression in clusters 2, 3, 4 and down regulation expression patterns in clusters 1, 5, 6, 7 and 8 at 72 hpi (Fig. 5b, Supplementary Table S4).

To identify the biological responses related to biochar-induced clusters, the up- and downregulation of eight co-expressed clusters of pathogen inoculated treatment at 72 hpi were evaluated for GO-enriched terms. The overrepresented GO-enriched terms in the biochar-upregulated clusters (2, 3, and 4) and down regulated clusters (1, 5, 6, 7 and 8) were evaluated using PANTHER GO analysis tool^24^. In cluster 2, enriched GO terms in biological process were related to cell wall biosynthesis and metabolic process, such as ‘xylan biosynthetic process’, ‘cellulose biosynthetic process’, ‘hemicellulose metabolic process’ ‘glucan biosynthetic process’, ‘pectin metabolic process’, ‘phenylpropanoid metabolic process’ cell wall polysaccharide biosynthetic process’ and more; enrichment in the molecular function category was also associated with responses to cell wall biosynthesis and metabolic process such as ‘cellulose synthase’, and more (Supplementary Table S5). In cluster 3, enriched GO terms in the biological process included ‘regulation of cell cycle’, ‘cell division’, ‘auxin-activated signaling pathway’, ‘cellular response to auxin stimulus’, ‘hormone-mediated signaling pathway’, ‘cellular response to hormone stimulus’, ‘cellular response to endogenous stimulus’, and more. Similarly, in cluster 4 enriched GO terms in the biological process category related to biotic stress and plant growth process, such as ‘regulation of response to stress’, ‘organic hydroxy compound biosynthetic process’, ‘regulation of jasmonic acid mediated signaling pathway’, ‘brassinosteroid biosynthetic process’, ‘brassinosteroid metabolic process’, ‘steroid biosynthetic process’, ‘sterol biosynthetic process’, ‘lignin biosynthesis and more (Supplementary Table S5). In cluster 1, enriched GO terms included ‘toxin metabolic process’, ‘iron ion binding’, ‘secondary metabolic process’, whereas in cluster 6 ‘inorganic anion homeostasis’ and ‘glycerolipid catabolic process’ were enriched GO terms. However, in cluster 5, 7 and 8 none of the GO terms were significantly enriched (Supplementary Table S5). To better understand the functions of these overrepresented GO terms, we characterized the metabolic and signaling pathways that were upregulated and downregulated (FDR < 0.05) in pathogen inoculated biochar treatment at 72 hpi.

### Differentially regulated metabolic and signaling pathways

KEGG mapper^25^ and Plant MetGenMAP^26^ were used to characterize the differentially regulated pathways only at 72 hpi using 2,249 transcripts of up- and downregulated transcripts that were assigned to 749 KEGG ID. Several pathways related to plant disease were systemically regulated by biochar in the presence of FORL: jasmonic acid, salicylic acid, ethylene, abscisic acid, sterol, and phenylpropanoid. Similarly, genes related to pathways modulating plant growth such as auxin, gibberellins, cytokinins, brassinosteroids, and others (cellulose, xylan) were also differentially regulated (Supplementary Table S6).

### Defense phytohormones

**Figure 6:**
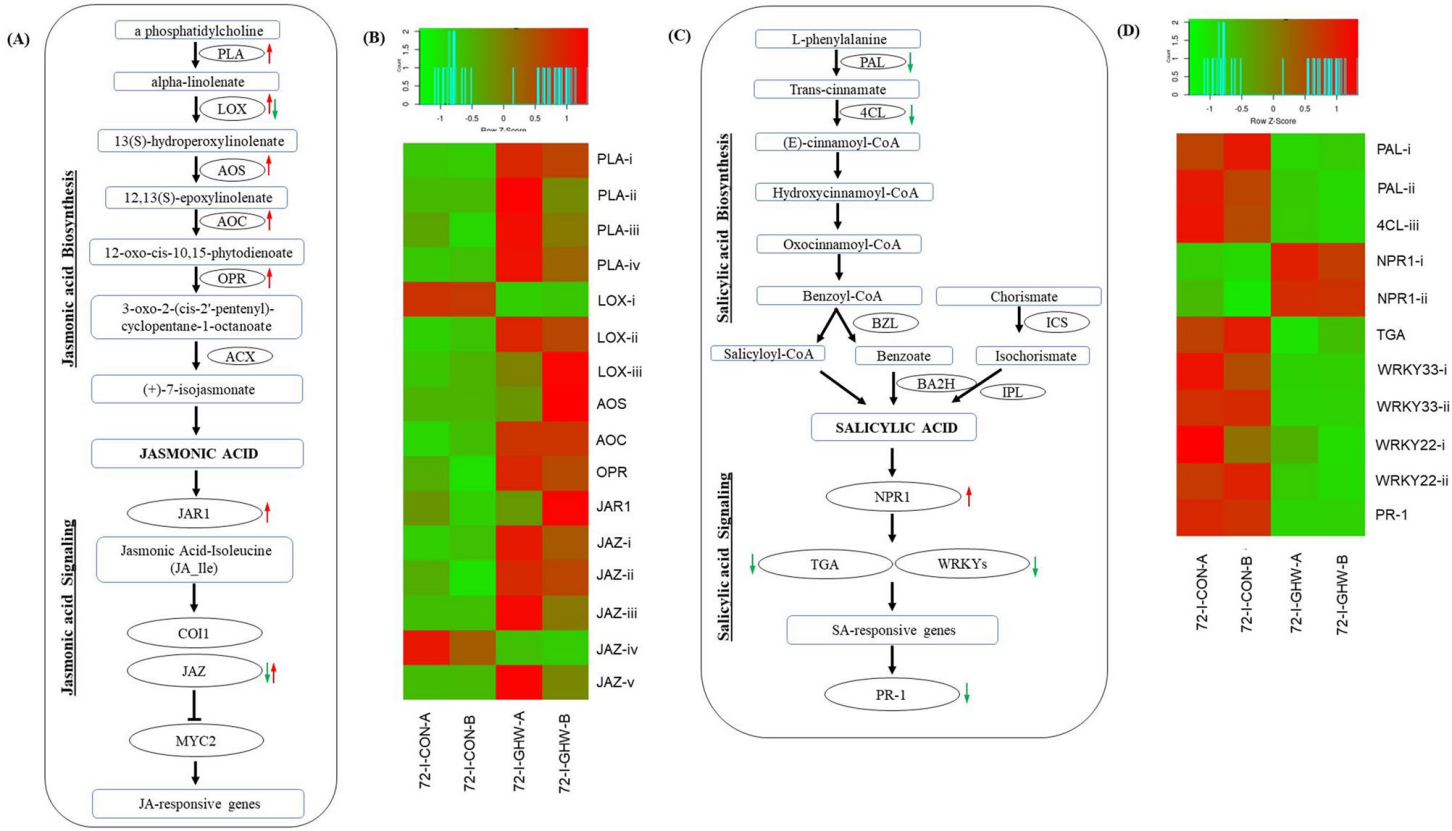
Jasmonic acid (JA) and Salicylic acid (SA) biosynthesis and signaling pathway systemically induced in response to biochar and pathogen at 72 hpi. (**A**) Plant induction of JA biosynthesis and signaling pathway. Transcripts marked with red and green arrow are significantly up- and downregulated, respectively; (**B**) Expression heatmap of transcripts related to JA biosynthesis and signaling in biochar amended and non-amended treatments at 72 hpi. Z-scores represent rescaled transcripts per kilobase per million (TPM) values; (**C**) Plant induction of SA biosynthesis and signaling pathway; (**D**) Expression heatmap of transcripts related to SA biosynthesis and signaling in biochar amended and non-amended treatments at 72 hpi. Abbreviations, transcripts identification, expression profile and FDR values are described in Supplementary Table S6.

JA biosynthesis and signaling pathways were systemically upregulated by biochar (Fig. 6a,b; Supplementary Table S6). Genes involved in JA biosynthesis including 4 phospholipases, 3 lipoxygenase (LOX), allene oxide synthase (AOS), allene oxide cyclase (AOC), and 12-oxophytodienoic acid reductase (OPR) were significantly upregulated (Supplementary Table S6). Similarly, genes involved in JA signaling including jasmonic acid-amido synthetase (JAR1-like) and 4 jasmonate ZIM-domain protein 1 (JAZ) were significantly upregulated; whereas also one JAZ was downregulated.

Nearly all genes involved in the SA biosynthesis and signaling were systemically downregulated by biochar (Fig. 6c,d; Supplementary Table S6), including two phenylalanine ammonia-lyase (PAL), 4-coumarate-CoA ligase (4CL), transcription factor TGA, two WRKY transcription factor 33, two WRKY transcription factor 22, and pathogen-related protein (PR1) (Supplementary Table S6). In contrast, regulatory protein two transcription factors of NPR1 were significantly upregulated by up to 3.5-fold.

Genes related to different steps of the ethylene biosynthesis and signaling transduction pathway had mixed expression patterns (Supplementary Fig. S1; Supplementary Table S6). S-adenosylmethionine synthase (SAMS) gene involved in the first step of ethylene biosynthesis was upregulated. Aminocyclopropane-1-carboxylate synthase 1/2/6 (ACS1_2_6) and aminocyclopropane-1-carboxylate synthase (ACS) were downregulated, whereas transcripts of 1-aminocyclopropane-1-carboxylate oxidase (ACO) were both upregulated and downregulated. In ET signaling pathway, ethylene-insensitive protein 3 (EIN3) and 4 ethylene-responsive transcription factor-1 (ERF1) were significantly downregulated (Supplementary Table S6). The genes involved in abscisic acid (ABA) biosynthesis and signaling were not significantly affected (data not shown).

### Phenylpropanoid and cell wall biosynthesis

**Figure 7:**
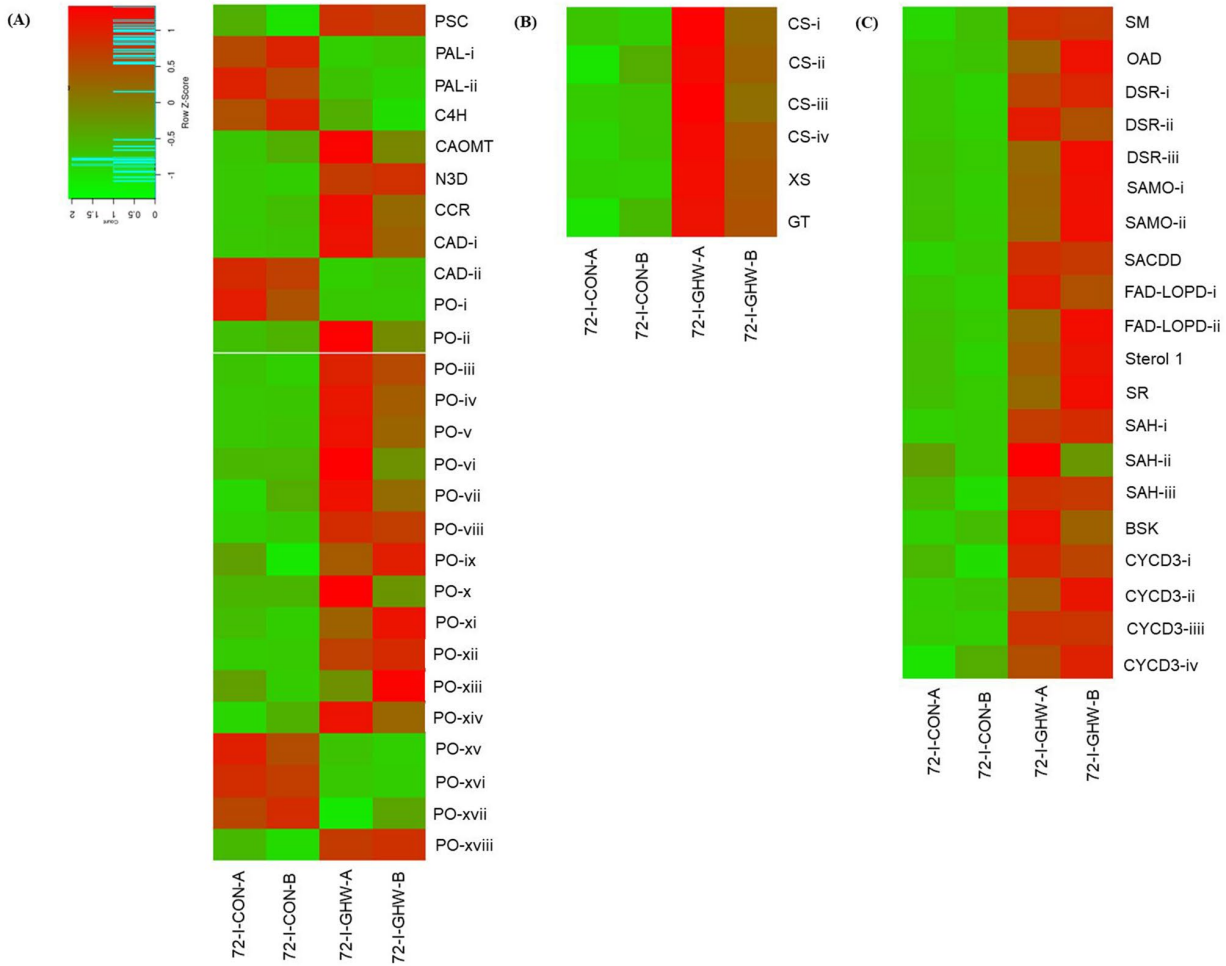
Phenylalanine, phenylpropanoid, flavonoid, lignin, cellulose, xylan, sterol and brassinosteroid biosynthesis pathway induced in response to biochar and pathogen at 72 hpi. Expression heatmap of genes related to induction of (**A**) phenylalanine, phenylpropanoid, flavonoid, and lignin biosynthesis, (**B**) cellulose and xylan biosynthesis, (**C**) sterol and brassinosteroid biosynthesis in biochar amended and non-amended treatments at 72 hpi. Z-scores represent rescaled transcripts per kilobase per million (TPM) values. Abbreviations, transcripts identification, expression profile and FDR values are described in Supplementary Table S6.

Phenylpropanoid, phenylalanine, flavonoid and lignin biosynthesis, which are known in defense response, were also significantly upregulated (Fig. 7a; Supplementary Fig. S2a, Supplementary Table S6). Genes activated in these pathways included 3-phosphoshikimate 1-carboxyvinyltransferase (PSC), caffeic acid o-methyltransferase (CAOMT), naringenin 3-dioxygenase (N3D), cinnamoyl-reductase (CCR), and two cinnamyl alcohol dehydrogenase-like protein (CAD). Upregulation of genes encoding for 14 peroxidases (PO), which play an important role in plant defenses against pathogens was also observed (Supplementary Table S6).

Secondary cell wall is mainly composed of cellulose, hemicelluloses (mostly xylans) and lignin, which play a crucial role in plant resistance to pathogens. Different genes involved in the cellulose and xylan were upregulated (Fig. 7b; Supplementary Fig. S2b, Supplementary Table S6). Key genes of cell wall synthesis, such as 4 cellulose synthase (CS), 1,4-beta-D-xylan synthase (XS) and Glycosyl transferase (GT) were upregulated (Supplementary Table S6).

### Sterol and Brassinosteroid biosynthesis

Overall biosynthesis of different forms of sterols (sitosterol, sigmasterol, brassicasterol, campesterol, crinosterol and cholesterol) were significantly upregulated (Fig. 7c; Supplementary Fig. S3a; Supplementary Table S6). Genes activated in this pathway included squalene monooxygenase (SM), obtusifoliol 14-alpha-demethylase or sterol 14-demethylase (OAD), 3 transcripts of delta14-sterol reductase (DSR), 2 transcripts of sterol 4-alpha methyl oxidase (SAMO), sterol-4alpha-carboxylate 3-dehydrogenase, decarboxylating (SACDD), 2 transcripts of flavin dinucleotide (FAD) linked oxidase domain protein (FAD-LOPD), sterol 1, and sterol reductase (SR) (Supplementary Table S6). The genes involved in the biosynthesis and signaling pathways of brassinosteroid, a phyto-steroidal hormone, and that has crucial roles in plant growth and disease resistance, were significantly upregulated by up to fivefold (Fig. 7c; Supplementary Fig. S2b; Supplementary Table S6). Those transcripts included 3 transcripts of steroid 5-alpha-reductase (SAH), BR-signaling kinase (BSK), and 4 transcripts of cyclin D3 (CYCD3) (Supplementary Table S6).

### Auxin, cytokinins and gibberellins

**Figure 8:**
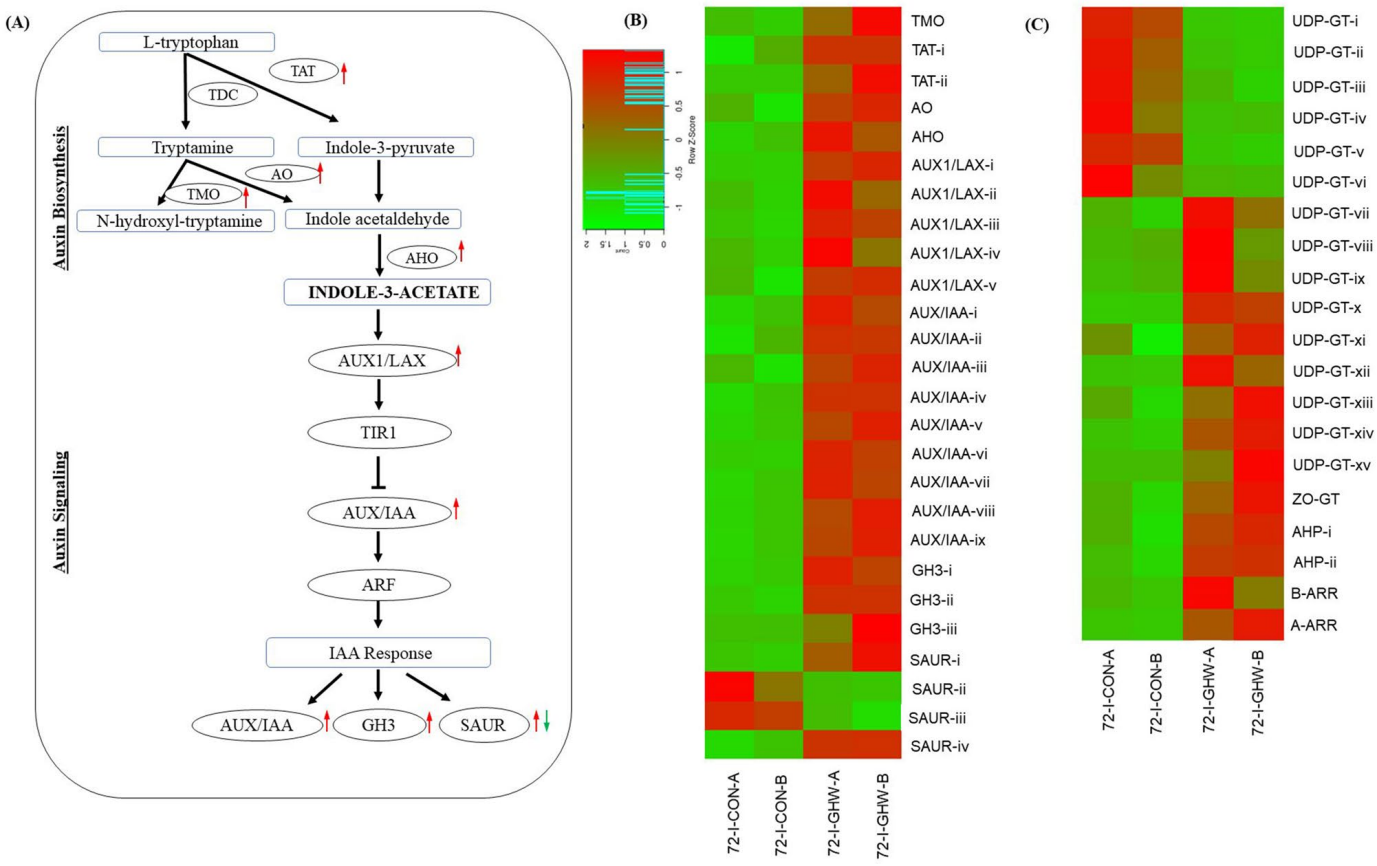
Auxin and cytokinins biosynthesis and signaling pathway systemically induced in response to biochar and pathogen at 72 hpi. (**A**) Plant induction of auxin biosynthesis and signaling pathway. Transcripts marked with red and green arrow are significantly up- and downregulated, respectively. Expression heatmap of transcripts related to (**B**) auxin and (**C**) cytokinins biosynthesis and signaling in biochar amended and non-amended treatments at 72 hpi. Z-scores represent rescaled transcripts per kilobase per million (TPM) values. Abbreviations, transcripts identification, expression profile and FDR values are described in Supplementary Table S6.

All genes involved in biosynthesis and signaling of indole-3-acetic acid (IAA), the main auxin in higher plants that is involved in the regulation of plant growth and development, are presented in Fig. 8a,b and Supplementary Table S6. Eight auxin-related genes were significantly upregulated. This included auxin biosynthesis genes: 2 transcripts of tryptamine monooxygenase (TMO), amine oxidase (AO), aldehyde oxidase (AHO) that were systemically upregulated by up to 8.3-fold. Auxin responsive genes were also significantly upregulated by up to 14.5-fold and the genes include: 5 transcripts of auxin influx carrier-AUX1 LAX family (AUX1/LAX), 9 transcripts of auxin-responsive protein IAA (AUX/IAA), 3 auxins responsive GH3, 2 small auxin-up RNA protein (SAUR). In contrast, two transcripts of SAUR were downregulated (Fig. 8a,b; Supplementary Table S6).

The pathway of cytokinin, a hormone that plays important role in several plant processes such as cell division and differentiation, was activated (Fig. 8c; Supplementary Table S6). Both biosynthesis and signaling pathways were upregulated by up to 12-fold, including nine transcripts of UDP-glucosyltransferase (UDP-GT), zeatin O-glucosyltransferase (ZO-GT), two histidine-containing phosphotransfer protein (AHP), two-component response regulator ARR-B family (B-ARR) and ARR-A family (Supplementary Table S6). Additionally, two key transcripts of gibberellin biosynthesis pathway (ent-kaurenoic acid oxidase; KAO) were upregulated but none of the signaling pathways were affected (Supplementary Table S6). Hence, plant growth hormones (auxin, cytokinin and gibberellin) were systemically upregulated by biochar.

### Validation by qRT-PCR

The expression pattern of differentially expressed genes identified in the RNA-Seq analysis was validated by qRT-PCR for 15 selected plant growth and defense-related genes (such as JA, SA, auxin, brassinosteroid biosynthesis and signaling, sterol and lignin biosynthesis genes listed in Supplementary Table S3). As the most significant changes in the gene expression and most of the enriched pathways appeared during the 72 hpi treatment, samples from this time were chosen. The results of qRT-PCR were very similar to those obtained by RNA-seq analysis across all treatments and all of the 15 analyzed genes (R^2^ = 0.8706; *P* < 0.0001, Supplementary Fig. S4 and Supplementary Table S7), indicating that RNA-seq correctly captured changes in gene expression.

## Discussion

In this study, we observed biochar-mediated systemic resistance against the soilborne pathogen FORL. In addition, we identified the importance of priming (i.e., “a faster and stronger expression of defense responses that become activated upon pathogen attack”^27^) for the systemic resistance, as well as the essential role of the pathogen in the expressed gene profile. Several systemic pathways that might be involved in the defense response were identified.

The temporal trends in gene expression patterns of inoculated biochar-treated compared with inoculated non-treated plants are evidence that the biochar treatment had a priming effect on gene expression upon infection. Priming is an integral part of both ISR and SAR, and is mediated by pathways that are dependent on JA, SA, abscisic acid, ethylene, ROS, and phenylpropanoids^28,29^. Indeed, many genes associated with the JA biosynthesis and signaling pathway were upregulated. JA is a lipid-derived signaling molecule that is involved in various plant developmental and defense processes^30,31^. The critical role for JA pathway in biochar-mediated protection against FORL was confirmed using additional experimental approach of JA-impaired *def1* plants. JA-dependent defenses mediated by biochar were also observed in tomato and strawberry against foliar pathogens^8,9^ and in rice against herbivores^32^. In contrast, Viger, et al.^21^ reported down-regulation of defense-related genes including JA in *Arabidopsis* grown in biochar. However, no pathogen challenge was made in their study and relatively high biochar concentrations of 4.2% (w:w) high temperature gasification biochar were used. As a result, it cannot be deduced whether the general downregulation of defense-involved genes would have resulted in subsequent susceptibility to pathogen attack. Excessive biochar concentrations (usually > 3%) have been seen to lead to increased plant susceptibility to pathogen attack, even though plant growth in the absence of the pathogen may not be negatively affected^11^.

In contrast, nearly all genes that are involved in the SA biosynthesis and signaling were downregulated except the regulatory protein NPR1, which was upregulated. The essential role of the transcriptional co-regulator NPR1 in SA-dependent SAR has been well characterized. NPR1 was shown to be required for JA/ET-dependent ISR triggered by PGPR and PGPF as well^14,33–35^. In SAR signaling, NPR1 functions as a transcriptional coactivator of SA-responsive PR genes and is clearly connected to a function in the nucleus^36^. In contrast, rhizobacteria-mediated JA/ET and ISR signaling typically functions without PR gene activation and is connected to cytosolic function^30,34,35,37^. Supporting our RNA-seq data, SA-deficient *NahG* indicated that SA signaling is not required for biochar-mediated protection against FORL. The observations that SA was activated in response to FORL in the biochar-free treatment, and that the SA-deficient *NahG* was more resistant to FORL, could lead to the conclusion that SA may have a negative effect on tomato plant resistance to the disease. These results demonstrate antagonistic crosstalk between the SA- and JA-defense pathways. Indeed, during immunity to necrotrophic pathogens, JA is needed, while the antagonistic action of SA could lead to susceptibility^30^.

JA is generally crucial to defense against pathogens with various life styles including *Fusarium*^38–40^. The phytohormone is involved in symbiotic interactions and moreover, it is well established that resistance induced by PGPR and PGPF is often JA-mediated^14,15,27,41,42^. Biochar amendment to soil has been shown to alter the composition of microbial communities and increase microbial diversity and activity in the bulk soil as well as in the plant rhizosphere^6,12,43^. Among the biochar-induced microorganisms, populations of PGPR and PGPF such as Fluorescent *Pseudomonas*, *Flavobacterium*, *Bacillus*, *Streptomyces strains* and *Trichoderma* spp. were significantly enhanced by the same biochar and in a similar pathosystem as the present study^12^. Jaiswal et al*.* (2017) also reported that biochar stimulated the growth of chitinolytic bacteria and cellulolytic bacteria. These bacteria digest fungal and oomycetes cell walls, releasing oligo-chitin and -glucan fragments which are known to be active elicitors of plant defense responses^44,45^.

Beside hormonal defense pathways, biochar induced the biosynthesis of cell wall and secondary metabolites. Secondary cell wall that is mainly composed of cellulose, hemicelluloses (mostly xylans) and lignin play a crucial role in plant resistance to pathogens^46^. Biochar upregulated different genes involved in cellulose and xylan biosynthesis. Similarly, some genes involved in phenylpropanoid, phenylalanine, flavonoid, and lignin biosynthesis were also significantly upregulated. These upregulated genes could increase the synthesis of ferulic acid, flavonoids (naringenin, kaempferol, quercetin), and lignin in plants, all of which can contribute to plant defense through antimicrobial activity and increasing cell wall firmness and stability^47–49^.

Peroxidases are widely known to play a central role in host plant defenses against pathogens^50^ and are involved in the lignin synthesis pathway, which is frequently responsive to JA or ET^51,52^. Peroxidases are expressed to limit cellular spreading of the infection through the establishment of structural barriers or the generation of highly toxic environments by massively producing reactive oxygen species (ROS) and reactive nitrogen species (RNS)^53^. Under severe stress, ROS production can exceed the scavenging capacity and accumulate to levels that can damage cell components, *e.g*., via lipid peroxidation^54^. Several beneficial microbes including *Trichoderma* spp. and *Pseudomonas florescence* have been shown to help protect plants against ROS, apparently by increasing their ability to scavenge ROS via increasing the production of detoxifying enzymes such as peroxidase, superoxide dismutase (SOD), glutathione-reductase and glutathione-S-transferase in leaves^55,56^.

Overall biosynthesis of different sterols (sitosterol, sigmasterol, brassicasterol, campesterol, crinosterol and cholesterol) was significantly upregulated in the biochar treatment under FORL inoculation. Sterols are structural components of the cell membranes and, together with sphingolipids, form the "lipid rafts" where enzyme and signaling complexes are localized^57^. Recently, sterol has been reported to be involved in induction of plant defense by *T. viride*^58^. In addition, sterols are precursors of the brassinosteroids, a group of plant hormones that regulate plant growth and development^57^ and have the potential to increase resistance to a wide spectrum of stress in plants^59^.

Improved plant performance by biochar was not associated with higher availability or acquisition of nutrients, as multi-element analysis of tomato plants revealed no change in nutrient contents or improvements in water holding capacity of the plant growing media between biochar amended and non-amended treatment^12^. However, in our current study we found a significant increase in expression level of biosynthesis and signaling of different plant growth promoting hormones. Biochar enriched microbes from genera such as *Pseudomonas*, *Bacillus* and *Trichoderma* in tomato rhizosphere and rhizoplane^12^, could have played a pivotal role in stimulating plant performance as these microbes often display the potential to produce or modulate plant growth hormones such as IAA, gibberellins, cytokinins, and ethylene^60–62^. Alternatively, biochar-borne chemicals (Supplementary Table S2) may have a hormone-like influence on plant growth as well as on plant defense.

Our data show evidence that auxin and brassinosteroids are central to biochar stimulated plant growth, supporting similar evidence observed previously^21^. Auxin is involved in almost every aspect of plant growth and development, such as shoot elongation, leaf growth, root growth and development, meristematic activity, root and shoot branching^63–65^. Recent studies have provided new insights into the role of auxin in plant defense against necrotrophs^66^ with synergistic interaction with JA^67^. For example, the axr1 mutant, related to auxin and JA signaling, was susceptive to the necrotrophic pathogen *Pythium irregulare*^68^. There is also an interplay between auxin-mediated plant growth and defense, for example, the tryptopan pathway produces auxin and defense-related antimicrobial secondary metabolites such as indole-glucosinolates and the phytoalexin camalexin^68,69^. Furthermore, auxin also interacts with brassinosteroids. For example, both auxin and brassinosteroids pathways synergistically regulate the expression of several auxin-responsive genes^70,71^.

The present work demonstrates the induction of systemic resistance in tomato against a soilborne pathogen by biochar, and elucidates some of the molecular pathways responsible for improved plant growth and enhanced plant defenses using whole transcriptomic analysis. The presence of biochar primed the plant for potentiated systemic responses to soilborne pathogen infection. In general, genes and pathways associated with plant defense and plant growth such as jasmonic acid, phenylpropanoids, flavonoid, peroxidases, sterol, brassinosteroids, auxin, cellulose, xylan, and lignin were upregulated. Understanding the mechanisms of biochar-mediated systemic resistance against pathogens and improved plant performance are important steps for the adoption of biochar as a beneficial soil amendment.

## Materials and methods

### Biochar and plant growing medium

GHW-350 biochar was produced at 350 °C highest treatment temperature (HTT) from greenhouse pepper plant wastes as previously described^12,72^. The most pertinent characteristics of the biochar are detailed in Supplementary Tables S1 and S2. A commercial potting mixture, peat: tuff (7:3 v:v mixture; Shaham Givat-Ada, Israel) was used as the plant growing medium.

### Plant growing condition, pathogen inoculation, and disease assessments

Tomato seeds (*S. lycopersicum*, cv. M-82, Zeraim Gedera, Israel) were sown after surface sterilization (with 1.5% NaOCl for five minutes followed by three times rinsing with sterile water) in a tray containing the potting mixture that was previously homogenized with or without GHW-350 biochar (0, 1, and 3% w:w). After germination, a single tomato seedling (21-day-old) was transplanted to each pot (0.5 L, diameter = 10 cm) containing potting mixture with or without biochar (0–3% w:w). FORL were added to the potting medium as previously described^12^. Briefly, FORL was cultured on pearl millet seeds (*Pennisetum glaucum*) that were previously soaked in water overnight and autoclaved twice on two consecutive days, 24 h apart. Twelve-day old FORL-infested millet seeds were mixed with the potting mixture at a concentration of 0.75% (w:w). Noninfected millet seeds which underwent the same autoclave preparation served as the non-inoculated control treatment. Each treatment included four biological replicates with five plants per biological replicate (total 20 plants per treatment). Transplanted seedlings were maintained in the glasshouse for 24 days at 22 ± 1 °C under fertigation and irrigation regimes as in previous studies^12^.

Five plants of each biological replicate were used to calculate the mortality rate per replicate, which was recorded daily until disease progress ceased. Daily mortality rate was used to draw disease progress curves and calculate the Area Under the Mortality Progress Curve (AUMPC in % × days), which represents the intensity of the entire epidemic. AUMPC was determined by the trapezoid method^3^. Plant growth and physiological parameters were evaluated at the end of the experiments (24 days after transplanting). Plant height was measured from stem base to top. Net photosynthesis rate were measured with a portable photosynthesis system (Li 6400XT, LI-COR Inc., Lincoln, NE)^73^.

### Systemic resistance to FORL in tomato seedlings

Five tomato genotypes were used to test the effect of biochar amendment on induced resistance against FORL: (1) commercial cv. M-82; (2) cv. Moneymaker and (3) its transgenic NahG plants that express the bacterial enzyme salicylate hydroxylase, which converts SA into biologically inactive catechol, resulting in plants deficient in SA accumulation^74,75^ (seeds were kindly provided by Yigal Cohen, Bar-Ilan University); (4) cv. Castlemart and (5) its JA-deficient mutant defenseless-1 (*def1*), which has a defect in the jasmonate pathway between 13-hydroperoxy-octadecatrienoic acid (13-HPOT) and 12-oxo-phytodienoic acid; this mutant fails to produce JA^76,77^ (seeds were kindly provided by Gregg Howe, Michigan State University). Surface sterilized seeds of these genotypes were sown in trays containing the potting mixture that was previously homogenized with or without GHW-350 biochar (0, 1, and 3% w:w). After germination, a single tomato seedling (7-day-old) was transplanted to each pot (0.5 L, diameter = 10 cm) containing potting mixture with or without biochar (0–3% w:w). Transplanted seedlings were maintained in a greenhouse for an additional 14 days at 22 ± 1 °C, under fertigation and irrigation regimes as in previous studies^12^.

In order to determine whether systemic induced resistance might be involved in disease suppression, a stem base inoculation approach was adopted to separate spatially the site of biochar treatment and the site of inoculation. An agar disc (3 mm) with an actively growing 5-day old colony of FORL was placed on stem base region (2–3 cm above soil) of 21-day-old tomato plant. A pathogen-free agar disc served as the non-inoculated control treatment (mock). All inoculation sites were immediately wrapped with parafilm and then covered with an aluminum foil to exclude light. Each treatment included four biological replicates with five plants per biological replicate (total 20 plants per treatment). Plants were inspected daily for disease measures.

### Transcriptome response to biochar and pathogen

#### Plant sampling

For the transcriptomic profiling study, the stem base inoculation system was adopted as described above in M-82 tomato genotype. Control stem base (pathogen-free) and FORL-inoculated stem base was collected at 0, 24- and 72-h post-inoculation (hpi). The collected stem base was snap-frozen in liquid nitrogen and stored at − 80 °C until use. Each biological sample included a pool of two individual plants and all together there were 20 biological samples, two replications from each of the following 10 treatments: (1) 0 hpi control (00-CON), (2) 0 hpi biochar (00-GHW); (3) 24 hpi control (24-NI-CON); (4) 24 hpi biochar (24-NI-GHW); (5) 72 hpi control (72-NI-CON); (6) 72 hpi biochar (72-NI-GHW); (7) 24 hpi control + FORL (24-I-CON); (8) 24 hpi biochar + FORL (24-I-GHW); (9) 72 hpi control + FORL (72-I-CON); and (10) 72 hpi biochar + FORL (72-I-GHW).

### RNA extraction, quality control and RNA-sequencing

Total RNA was extracted from tomato stem base samples (200 mg) using the GenElute mammalian total RNA miniprep kit (Sigma-Aldrich, USA) according to the manufacturer’s protocol, with one modification: tissue was ground in lysis buffer and mercaptoethanol with two 0.5-cm-diameter tungsten balls using FastPrep-24 5G Instrument (MP Biomedicals, Santa Ana, California, USA) at 6.0 m/s for 40 s for two cycles. The samples were placed in ice for 2 min between cycles. Extracted RNA was treated with DNase (TURBO DNA-free Kit, Ambion Life Technologies, USA) to remove possible genomic DNA traces. RNA yield and purity were measured by Nanodrop (ND-1000 Spectrophotometer, Wilmington, USA) and integrity by running in 1.5% agarose gel electrophoresis. Furthermore, RNA was validated for quality by running an aliquot on a Bioanalyzer 2,200 Tape-Station (Agilent Technologies, California, USA). The cDNA libraries were prepared for sequencing using TrueSeq RNA kit (Illumina, San Diego, CA, USA). Libraries were evaluated with Qubit and TapeStation (Agilent Technologies, California, USA) and sequencing libraries were constructed with barcodes to enable sample multiplexing. Pooled libraries of the 20 samples were sequenced on two lanes (to obtain 10–20 million reads per sample at end) of an Illumina Hiseq 2,500 instrument using a 60-bp single-end RNA-Seq protocol at the Nancy and Stephen Grand Israel National Center, Weizmann Institute of Science, Israel.

### Bioinformatics analysis of RNA-Seq data

Quality of Illumina sequencing was checked with FASTQC (https://www.bioinformatics.babraham.ac.uk/projects/fastqc/). The raw reads were subjected to quality trimming and filtering, and adapter removal by Trimmomatic software^78^. Cleaned sequences were mapped to a tomato reference (ITAG 3.10_cDNA) using bowtie2 software^79^. RSEM software^80^ was used to calculate abundance estimates for each tomato transcript. DESeq2^81^ of the Bioconductor R packages^82^ was used to identify differentially expressed transcripts for each biological replicate, based on the count estimates for each transcript. Transcript counts were normalized by trimmed mean of M values (TMM)^83^ and differentially expressed genes were calculated using DESeq2 R package^81^. The sequence data generated in this study was submitted to the NCBI under bioproject accession number PRJNA515188. The genes were annotated by BLASTx^84^ against the non-redundant NCBI protein database, after which their gene ontology (GO) term^85^ was assigned by combining BLASTx data and interproscan analysis^86^ by means of the BLAST2go software pipeline^87^. Genes that were expressed greater than or less than log2fold + 1 or − 1, respectively, with a FDR (False Discovery Rate) of the p value less than 0.05, were considered differentially expressed. The expression patterns of the genes of inoculated treatments at different time points were studied using cluster analysis of differentially expressed genes in at least one pairwise biological replicate comparison. Expression normalization was calculated using trimmed mean of M-values. Then, hierarchical clustering of genes and biological replicates was performed and clusters were extracted using hierarchical clustering based on Euclidean distance matrix (with the R scripts hclust function). Principal component analysis (PCA) and 2D hierarchical clustering were performed on normalized data using R package ‘FactomineR’^88^. GO terms classification and GO enrichment analysis by Fisher’s exact test with multiple testing correction of FDR (< 0.05) of upregulated or downregulated genes clusters was carried out by using PANTHER GO analysis tool^24^. Distribution of transcripts into various biological pathways in Kyoto Encyclopedia of Genes and Genomes (KEGG) was done through the KEGG Automatic Annotation Server (https://www.genome.jp/tools/kaas/) to obtain the KEGG IDs for the transcriptome sequences, and to identify the genes involved in plant hormone signal transduction^89^. Pathways analysis of upregulated and downregulated genes was performed using KEGG mapper (https://www.genome.jp/kegg/mapper.html) and Plant MetGenMAP^26^.

### Validation with qRT-PCR

The expression pattern of differentially expressed genes identified in the RNA-Seq analysis was validated by relative quantification of selected 15 plant growth and defense-related genes expressed at 72 hpi using StepOnePlus Real-Time PCR instrument (Applied Biosystems, USA). The role of the q-PCR analysis was to provide more evidences to the involvement of specific pathways and to confirm the accuracy of the RNA-seq with additional method. The primers for each of the 15 genes were designed by the software PRIMER3 (https://bioinfo.ut.ee/primer3-0.4.0/) (Supplementary Table S3). Each PCR amplification was performed for three independent biological repeats with two technical repeats in a 15 µl reaction mix containing 7.5 µl 2 × ABsolute SYBR Green Rox Mix (Thermo Scientific), 1 µl each of the forward and reverse primers (3 µM), 4 μl cDNA (diluted 1:4; 20 µl cDNA + 60 µl ultra-pure water), and 1 µl PCR-grade water. The PCR program consisted of an initial denaturation at 95 °C for 10 min, followed by 40 cycles of 10 s at 95 °C, 15 s at 60 °C and 20 s at 72 °C. Relative quantification was performed by the ΔΔC_T_ method^90^. The ΔC_T_ value was determined by subtracting the C_T_ results for the target gene from the endogenous control gene-TIP41^91^ and ribosomal protein L2 (RPL2)^92^ and normalized against the calibration sample to generate the ΔΔCT values. In order to check amplification efficiency or factor of a PCR reaction, standard curves based on Ct values vs. log (cDNA dilution) were constructed using serial tenfold dilution of cDNA for each pair of selected primers. The sequences of all primers and their amplification factors are outlined in Table S3. The results of the expression levels of the genes with the qRT-PCR and those of the RNA-seq were correlated with a linear regression.

## Supplementary information

Supplementary figures

Supplementary tables
