# Supplementary material for: Molecular insights into biochar-mediated plant growth promotion and systemic resistance in tomato against Fusarium crown and root rot disease
Source: Sci Rep. 2020 Aug 18;10:13934. doi: 10.1038/s41598-020-70882-6 (PMC7434890; doi:10.1038/s41598-020-70882-6)
Supplement: Supplementary file 1 — Supplementary figures [file 41598_2020_70882_MOESM1_ESM.docx]

**Supplementary Information**

**Molecular Insights into Biochar-Mediated Plant Growth Promotion and Systemic Resistance in Tomato against Fusarium Crown and Root Rot Disease**

*Amit K. Jaiswal, Noam Alkan, Yigal Elad, Noa Sela, Amit M. Philosoph, Ellen R. Graber, Omer Frenkel*

**Supplementary Figure S1:** Ethylene (ET) biosynthesis and signaling pathway systemically induced in response to biochar and pathogen at 72 hpi. (a) Plant induction of ET biosynthesis and signaling pathway. Transcripts marked with red and green arrow are significantly up- and downregulated, respectively. (b) Expression heatmap of transcripts related to ET biosynthesis and signaling in biochar amended and non-amended treatments at 72 hpi. Z-scores represent rescaled transcripts per kilobase per million (TPM) values. Abbreviations, transcripts identification and expression profile are described in Supplementary Table S5.

**Supplementary Figure S2:** Phenylalanine, phenylpropanoid, flavonoid, lignin, cellulose, and xylan, biosynthesis pathway induced in response to biochar and pathogen at 72 hpi. Plant induction of (a) phenylalanine, phenylpropanoid, flavonoid and lignin biosynthesis pathway, (b) cellulose, and (c) xylan biosynthesis pathway. Transcripts marked with red and green arrow are significantly up- and downregulated, respectively. Abbreviations, transcripts identification and expression profile are described in Supplementary Table S5.

**Supplementary Figure S3:** Sterol and brassinosteroid biosynthesis and signaling pathway induced in response to biochar and pathogen at 72 hpi. Plant induction of (a) sterol and (b) brassinosteroid biosynthesis and signaling pathway. Transcripts marked with red and green arrow are significantly up- and downregulated, respectively. Abbreviations, transcripts identification and expression profile are described in Supplementary Table S5.

**Supplementary Figure S4:** Correlation analysis of gene expression values obtained from RNA-seq and qRT-PCR analysis
